# Devil in the details? Developmental dyslexia and visual long-term memory for details

**DOI:** 10.3389/fpsyg.2014.00686

**Published:** 2014-07-02

**Authors:** Lynn Huestegge, Julia Rohrßen, Muna van Ermingen-Marbach, Julia Pape-Neumann, Stefan Heim

**Affiliations:** ^1^Institute of Psychology, Würzburg UniversityWürzburg, Germany; ^2^Institute of Psychology, RWTH Aachen UniversityAachen, Germany; ^3^Department of Psychiatry, Psychotherapy and Psychosomatics, Uniklinik RWTH AachenAachen, Germany; ^4^SRH University of Applied Sciences for HealthGera, Germany; ^5^Section Clinical and Cognitive Neurosciences, Department of Neurology, Medical School, RWTH Aachen UniversityAachen, Germany; ^6^Institute of Neuroscience and Medicine (INM-1), Research Centre JülichJülich, Germany; ^7^JARA – Translational Brain MedicineJülich and Aachen, Germany

**Keywords:** picture processing, memory errors, orthographic representations, visual resolution deficit, phonology and reading, language and word processing

## Abstract

Cognitive theories on causes of developmental dyslexia can be divided into language-specific and general accounts. While the former assume that words are special in that associated processing problems are rooted in language-related cognition (e.g., phonology) deficits, the latter propose that dyslexia is rather rooted in a general impairment of cognitive (e.g., visual and/or auditory) processing streams. In the present study, we examined to what extent dyslexia (typically characterized by poor orthographic representations) may be associated with a general deficit in visual long-term memory (LTM) for details. We compared object- and detail-related visual LTM performance (and phonological skills) between dyslexic primary school children and IQ-, age-, and gender-matched controls. The results revealed that while the overall amount of LTM errors was comparable between groups, dyslexic children exhibited a greater portion of detail-related errors. The results suggest that not only phonological, but also general visual resolution deficits in LTM may play an important role in developmental dyslexia.

## INTRODUCTION

Reading disabilities pose a huge risk to a successful individual development in our society and can have far-reaching academic, occupational, and psycho-social consequences. A substantial body of research has been accumulated to understand the scope and causes of reading problems. Most of this research has been devoted to developmental dyslexia, which is usually characterized as a selective deficit in reading abilities that is unexpected in relation to age, other cognitive skills, and learning opportunities (Cain, 2010). Developmental dyslexia is reported to affect about 5–15% of the population (Vellutino et al., 2004). It is associated with a male preponderance (Thambirajah, 2011), and typically displays lifelong persistence (Ramus, 2003).

Several potential causes of developmental dyslexia have been discussed, ranging from language-specific to general cognitive deficits. Probably the most prominent language-specific account is the phonological deficit hypothesis (Vellutino, 1979; Snowling, 2000, 2001; Bishop and Snowling, 2004; for a discussion see Ramus, 2003). According to this claim, reading problems are based on specific deficits in phonological awareness, a concept associated with representing, storing, processing, and retrieving basic speech sounds (or phonemes), as well as mapping letters to speech sounds. The phonological deficit account is strongly supported by the fact that tasks requiring phonological awareness (e.g., rhyming tasks, phoneme substitution tasks) represent one of the best predictors of reading problems (Goswami and Bryant, 1990), along with tasks involving speeded access to phonological word forms in rapid automatized naming of letters or digits (Denckla and Rudel, 1976; Wolf and Bowers, 1999).

As opposed to language-specific accounts, more general cognitive theories assume that phonological deficits, as well as other symptoms, may be based on general auditory processing deficits (Tallal et al., 1993; Boets et al., 2007; but see Studdert-Kennedy, 2002), or even on supra-modal (i.e., auditory and visual) deficits in temporal processing (see Farmer and Klein, 1995). Other general theories of dyslexia place stronger emphasis on biological roots of impairments. For example, the cerebellar deficit theory assumes that dyslexic symptoms are based on cerebellar dysfunctions that eventually impair automaticity of cognitive and motor skills relevant for reading (Nicolson et al., 1995). From this perspective, dyslexia may actually constitute a procedural learning deficit (Nicolson and Fawcett, 2011). More recently, the magnocellular deficit theory was proposed (Stein, 2001), partly based on observations that dyslexics exhibit lower visual contrast and motion sensitivity. According to this theory, deficits in the magnocellular layers in the brain may lead to unstable visual fixation, vergence problems, and difficulties in encoding letter sequences, among other deficits in auditory and motor domains (Stein and Walsh, 1997; but see Olulade et al., 2013).

Finally, another branch of research on dyslexia specifically focuses on visual processing. For example, many studies examined the role of visual working memory, suggesting that reading deficits were associated with lower performance in verbal working memory only, not visual working memory in general (Vellutino, 1979; Hulme, 1981; Katz et al., 1981; McDougall et al., 1994; Scanlon and Vellutino, 1997; but see Smith-Spark et al., 2003; Menghini et al., 2011; Huestegge et al., 2012). However, recent research that focussed on visual attention rather than on visual working memory has led to a revival of the idea that impaired visual processing in general may be causally related to developmental dyslexia. For example, Vidyasagar and Pammer (2010) suggested that deficits in visuo-spatial attention may impair letter sequence processing, so that impoverished orthographic information is fed into word recognition systems (see also Valdois et al., 2004; Bosse et al., 2007; Jones et al., 2008; for related visual accounts). In line with a general cognitive account of reading it should also be noted that reading as a cultural technique is too young to be based on dedicated genetic mechanisms. In fact, recent theories on reading development posit that brain circuits originally evolved for object recognition may become tuned to recognize frequent letters and words (Dehaene and Cohen, 2007).

The apparent diversity of explanatory accounts of dyslexia may partly be rooted in the vast array of dyslexic symptoms, but also touches on the complexity of the task of reading, which involves visual, orthographic, phonological, semantic, and syntactic processing. Generally speaking, reading for comprehension is essentially characterized by transforming complex visual patterns (i.e., letters and words) into meaning (and/or spoken language) by mapping visual input to representations stored in long-term memory (LTM). Research on reading aloud has suggested two main processing routes, namely a grapheme-to-phoneme conversion stream (non-lexical route) and a lexical pathway that relies on the visual word form to gain lexical access (orthographic lexicon). For pronunciation purposes, the lexical route also involves access to a phonological lexicon further downstream (Coltheart et al., 2001).

Phonological accounts and visual attention accounts of dyslexia partly differ in their localization of deficits along these two routes. The former predict that dyslexics should encounter problems with grapheme–phoneme conversion (during oral reading) within the non-lexical route (especially for beginning readers), but also downstream on the lexical route where the phonological output lexicon is entered for pronunciation purposes. While visual attention deficits could principally also impair grapheme–phoneme conversion in non-lexical processing, their impact on lexical processing should rather be more upstream on the level of visual word form processing (access to orthographic lexicon). Visual attention problems may also provide a more convincing explanation of reading deficits during silent (vs. oral) reading. Empirical studies on the role of these processing routes in dyslexia yielded evidence for most of these assumptions (Ziegler et al., 2008; Hawelka et al., 2010).

While the majority of research on dyslexia in the past has focused on phonological processing, the process of matching visual input to stored orthographic representations in LTM has received comparatively little attention. Consequently, it appears especially important to further study visual processing and its relation to LTM (Dehn, 2010) in dyslexia. Since the visual attention deficit account maintains that dyslexics suffer from processing impoverished visual input, this should eventually also lead to degraded orthographic representations in the mental lexicon. This process may instantiate a vicious circle because poor representations in LTM should in turn make it harder to decipher new visual input. Given that words may only be special cases of highly detailed visual objects and that brain circuits involved in written language recognition are closely related to object recognition in general (Dehaene and Cohen, 2007), the question arises whether general resolution deficits in visual LTM (VLTM) may be associated with developmental dyslexia. The present study aims at providing a first step to put this hypothesis to test.

More specifically, we aimed at resembling the process of establishing orthographic representations by utilizing language-free visual material. A key feature of orthographic representations (as opposed to representations of many other visual objects) is the precise storage of miniscule details, given that small changes of visual features in letter strings can cause fundamental differences in meaning (e.g., “a mother” vs. “another”; “fail” vs. “tail”). Thus, we speculated that a deficit in VLTM for details (VLTM-D) may contribute to poor orthographic matching processes between visual word input and orthographic representations in LTM.

Previous studies on the role of LTM in dyslexia yielded rather mixed results. For example, Nelson and Warrington (1980) reported normal LTM functions in developmental dyslexia (see also Bell, 1990). In contrast, a recent Italian study reported specific LTM impairments in dyslexics (Menghini et al., 2010; see also Watson and Willows, 1995). Probably, these heterogeneous findings are due to the fact that those studies did not explicitly address resolution issues in LTM, that is, the ability to acquire highly detailed representations of visual objects.

**FIGURE 1:**
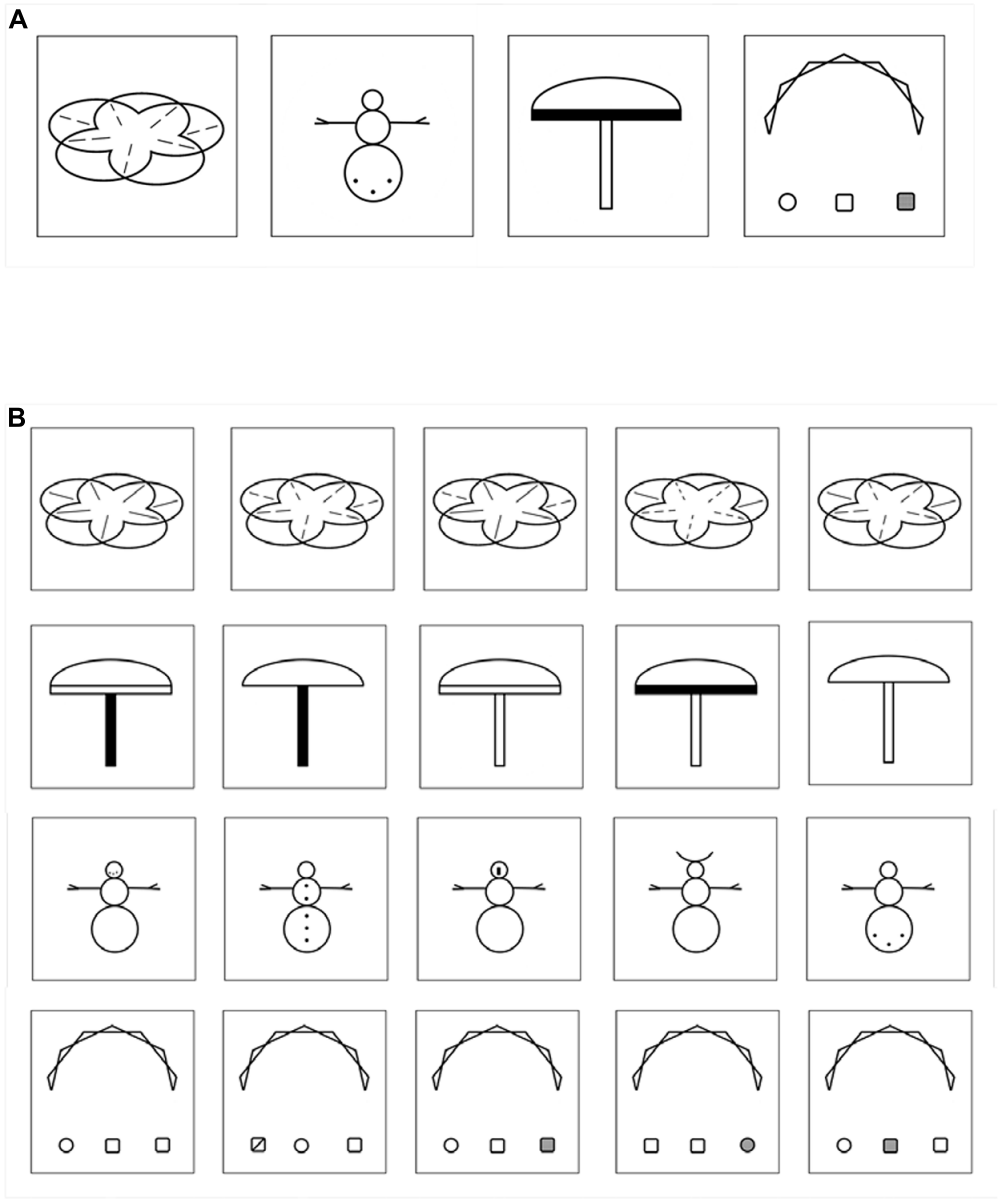
**(A)** Four examples of VLTM-D items presented in the encoding phase (5 s each). **(B)** Four examples of item arrays presented in the recognition phase. Each array consists of five items differing in small details, only one item was previously presented in the encoding phase. Note that half of the item arrays presented in the recognition phase did not resemble any item presented in the encoding phase and thus required a “no” response.

In a previous study, Huestegge et al. (2012) already utilized a VLTM-D task to study gender differences in a sample of normally developed British primary school children (i.e., without dyslexia). The VLTM-D task entails the memorization and (later on) the recognition of abstract but highly detailed geometrical figures (see **Figure 1**). Resulting variables include overall memory errors, but also include a score specifically for detail-related LTM. Note that in this previous study, there was no evidence for a direct association between VLTM resolution and reading abilities. However, this might have been due to the absence of participants with reading problems.

In the present study, we explicitly compared normal children with age-, IQ-, and gender-matched dyslexics to directly address the question of whether developmental dyslexics exhibit specific VLTM-D deficits (beside deficits in phonological processing). As reasoned above, a deficit of establishing highly resolved (i.e., detailed) VLTM representations (as opposed to more coarse object-based LTM representations) in dyslexics could represent a general, basic cognitive mechanism underlying problems regarding the establishment of solid (and sufficiently detailed) orthographic long-term representations, eventually causing reading problems. Based on this reasoning, we predicted a general spatial resolution deficit in VLTM of dyslexic children.

## MATERIALS AND METHODS

### PARTICIPANTS

All participants were German primary school students (native speakers at the end of grade three or beginning of grade four). Informed consent was obtained from all children, their parents, and school headmasters. Additionally, we received approval of the current study by the internal review board (ethics committee) of the Medical Faculty of RWTH Aachen University. Specifically, we tested 21 dyslexic children (*M* = 112 months, SD = 5) and 21 controls (*M* = 112 months, SD = 4). Since gender, age, and intelligence (IQ) are known to affect reading and memory abilities (e.g., Gathercole, 2003; Hales, 2008; Huestegge et al., 2012), groups were carefully matched regarding these variables (see **Table 1**), all *p*s > 0.80. For this matching procedure, we first tested the dyslexic participants. Then, we tested normal children until we arrived at sufficiently matched samples. This procedure necessarily involved the assessment of more children than reported here, but it is important to note that the ultimate sample selection was solely completed in the face of the matching variables (i.e., without any knowledge of the dependent variables or outcomes of statistical tests in order to avoid cherry-picking).

**Table 1:** Participant characteristics as a function of group.

	Dyslexics (*n* = 21)		Controls (*n* = 21)	
	female = 8, male = 13		female = 8, male = 13	
	*M*	SD	*M*	SD
Age (months)	112	4.06	112	5.20
Non-verbal IQ (CFT 20)	115	9.64	115	14.92
Reading quotient (SLS)	79	9.52	113	15.86
Phonological ability (subtest 1)	52	8.14	56	6.57
Phonological ability (subtest 2)	45	7.93	55	5.95

Developmental dyslexia in the dyslexic group was diagnosed by trained speech-language therapists from the local university hospital. We confirmed the presence of a strong difference in reading abilities between groups by implementing the following inclusion criteria for the dyslexic group: a non-verbal IQ score greater than 85 [as assessed using the Cultural Fair Test (CFT 20), see below], a reading score of at least one standard deviation below the mean in a widespread German screening test for reading abilities Salzburger Lese-Screening (SLS; Mayringer and Wimmer, 2003, see below), and performance of at least one standard deviation below the mean in at least one of the subtests of the German “Knuspel” test for reading abilities (Marx, 1998), the latter being an in-depth assessment of reading abilities including recoding/decoding on word level and reading comprehension. These diagnostic procedures were run in order to establish two age-, gender-, and IQ-matched groups of children with substantial differences in reading abilities.

Note that a strong test for a causal role of VLTM-D deficits for dyslexia would actually necessitate a longitudinal (or, with some reservation, a reading-age matched) design (Goswami, 2003). However, given the lack of any previous data on the association of VLTM-D and dyslexia, we opted for the present age-matched design as a first step toward testing for a connection between dyslexia and VLTM-D performance (see also General Discussion).

### MATERIAL AND PROCEDURE

Children were tested individually in a quiet and separate room in school. Each assessment lasted about 60 min.

#### Visual long-term memory for details

At the beginning of each test session, the encoding part of the VLTM-D task (Huestegge et al., 2012) was administered. Specifically, children were instructed to memorize 25 complex abstract (black and white) figures that were presented on a computer screen for 5 s each (see **Figure 1A**, for examples). After an interval of 45–60 min (which was used for implementing the remainder of assessments, see below) the recognition phase of the VLTM-D task was administered. Children were presented with 50 arrays of items, each array depicting five figures differing in small visual details (see **Figure 1B**, for examples). Half of the arrays contained one item that was previously presented during the encoding period. The children’s task was to indicate (a) if *any* of the five items was present in the encoding phase (object-related question, 50% guessing probability) and (b) if yes, which one exactly (detail-related question, 20% guessing probability conditional upon correct answer to a). This task provides three different types of scores: (1) total number of errors (wrong answer given in either the object-related or the detail-related question), (2) number of object-related errors (including false alarms and misses), and (3) number of detail-related errors (the latter two summing up to the total number of errors). Note that the object- and detail-related error categories are not statistically independent, since detail-related errors are only possible when no object-related error is made (thus, signal detection analysis appears unfeasible). To account for statistical dependency, two variables were finally computed and statistically analyzed, namely the “total percentage of memory errors” (based on the sum of both object- and detail-related errors), and the “portion of detail-related errors,” indicating the percentage of detail-related errors relative to the total number of errors. Note that equal probabilities of response alternatives for both questions ensured an interpretation of accuracy data uncompromised by potential response biases.

#### Reading abilities

All children were tested using the SLS (Mayringer and Wimmer, 2003), which measures reading speed and basic reading comprehension. Children silently read simple sentences (e.g., “Strawberries are blue”) and indicate whether the statement is correct or not. Scores are based on the number of correctly judged sentences within 3 min and transformed into reading quotients using grade- and age-specific norms.

#### General cognitive (non-verbal) abilities

For assessing non-verbal reasoning abilities, part one (including four subtests) of the CFT 20 (Cattell, 1960; revised by Weiss, 1998) was administered. Performance was converted into non-verbal IQ scores using age-specific norms.

#### Phonological awareness

To assess phonological awareness we administered the German BAKO 1–4 (Stock et al., 2003). Due to time constraints only the first two subtests were selected. In the first subtest, “pseudo-word segmentation,” children named phonemes of words which were aurally presented through a computer. In the second subtest, “vowel substitution,” children were asked to substitute all incidences of phoneme /a/ for phoneme /i/ in a series of aurally presented words and to pronounce the resulting new (pseudo-)words (e.g., HAMMER – HIMMER). The number of errors in the two subtests was transformed into *T*-scores based on age-specific norms.

### DATA ANALYSIS

Data were analyzed by computing a multivariate analysis of covariance (MANCOVA) to account for the statistical dependency among the dependent variables. While group (controls vs. dyslexics) served as the independent variable, gender, age, and IQ were added as covariates. Four measures served as dependent variables in the model, namely total percentage of visual memory errors, portion of detail-related visual memory errors, pseudo-word segmentation skills (BAKO subtest 1), and vowel substitution skills (BAKO subtest 2). For a more detailed analysis of the effects on the individual dependent variables, the MANOVA was followed up by separate univariate ANOVAs. The comparison of reading abilities (SLS scores) between groups, which essentially represented a manipulation check of the group variable, was based on a *t*-test for independent samples.

## RESULTS

In the SLS, dyslexic children exhibited a significantly lower reading score (*M* = 79; SE = 2.1) as compared to controls (*M* = 113; SE = 3.5), *t*(40) = 8.49, *p* < 0.001 (see **Table 1**). All children in the control group exhibited reading scores over 80, ensuring that there was no dyslectic child in the control group (see Mayringer and Wimmer, 2003, for a corresponding criterion).

The MANCOVA included the independent variable group (controls vs. dyslexics), the covariates gender, age, and IQ, and the dependent variables total percentage of memory errors, portion of detail-related errors, pseudo-word segmentation skills (BAKO subtest 1), and vowel substitution skills (BAKO subtest 2). Box’s *M*-test revealed no significant equality violation of the covariance matrices of the dependent variables across groups. As a result, the multivariate test revealed no significant effects of the covariates age and gender, both *F* < 1, but a marginally significant effect of the covariate IQ, *F*(4,34) = 2.54, *p* = 0.058, $\eta_{p}^{2}$ = 0.23. Crucially, there was a significant group effect, *F*(4,34) = 7.38, *p* < 0.001, $\eta_{p}^{2}$ = 0.47 (see **Table 2**).

**Table 2:** Multivariate analysis of covariance results for the covariates and the group comparison.

Effect	Wilks’s λ	*F*(4,34)	*p*	$\eta_{p}^{2}$
Gender (covariate)	0.97	0.31	0.871	0.035
Age (covariate)	0.92	0.78	0.544	0.084
IQ (covariate)	0.77	2.54	0.058	0.230
Group (controls vs. dyslexics)	0.54	7.38	<0.001	0.465

Following up on the multivariate analysis, we computed univariate ANOVAs to specify the individual impact of the covariate IQ and the group variable on the individual dependent variables (see **Table 3**). As a result, IQ significantly affected vowel substitution skills (BAKO subtest 2), *F*(1,37) = 5.54, *p* = 0.024, $\eta_{p}^{2}$ = 0.13, but none of the other dependent variables, all *F* < 1.4.

**Table 3:** Analysis of variance results for the (marginally significant) covariate IQ and the group factor (see MANCOVA results).

Effect	Dependent variable	*F*(1,37)	*p*	$\eta_{p}^{2}$
IQ (covariate)	Total percentage of visual memory errors (VLTM-D)	1.38	0.248	0.036
	Portion of detail-related memory errors (VLTM-D)	0.19	0.668	0.005
	Pseudo-word segmentation (phonological ability, subtest 1)	1.33	0.256	0.035
	Vowel substitution (phonological ability, subtest 2)	5.54	0.024	0.130
Group (controls vs. dyslexics)	Total percentage of visual memory errors (VLTM-D)	0.90	0.349	0.024
	Portion of detail-related memory errors (VLTM-D)	4.20	0.048	0.102
	Pseudo-word segmentation (phonological ability, subtest 1)	3.87	0.057	0.095
	Vowel substitution (phonological ability, subtest 2)	22.30	<0.001	0.376

**FIGURE 2:**
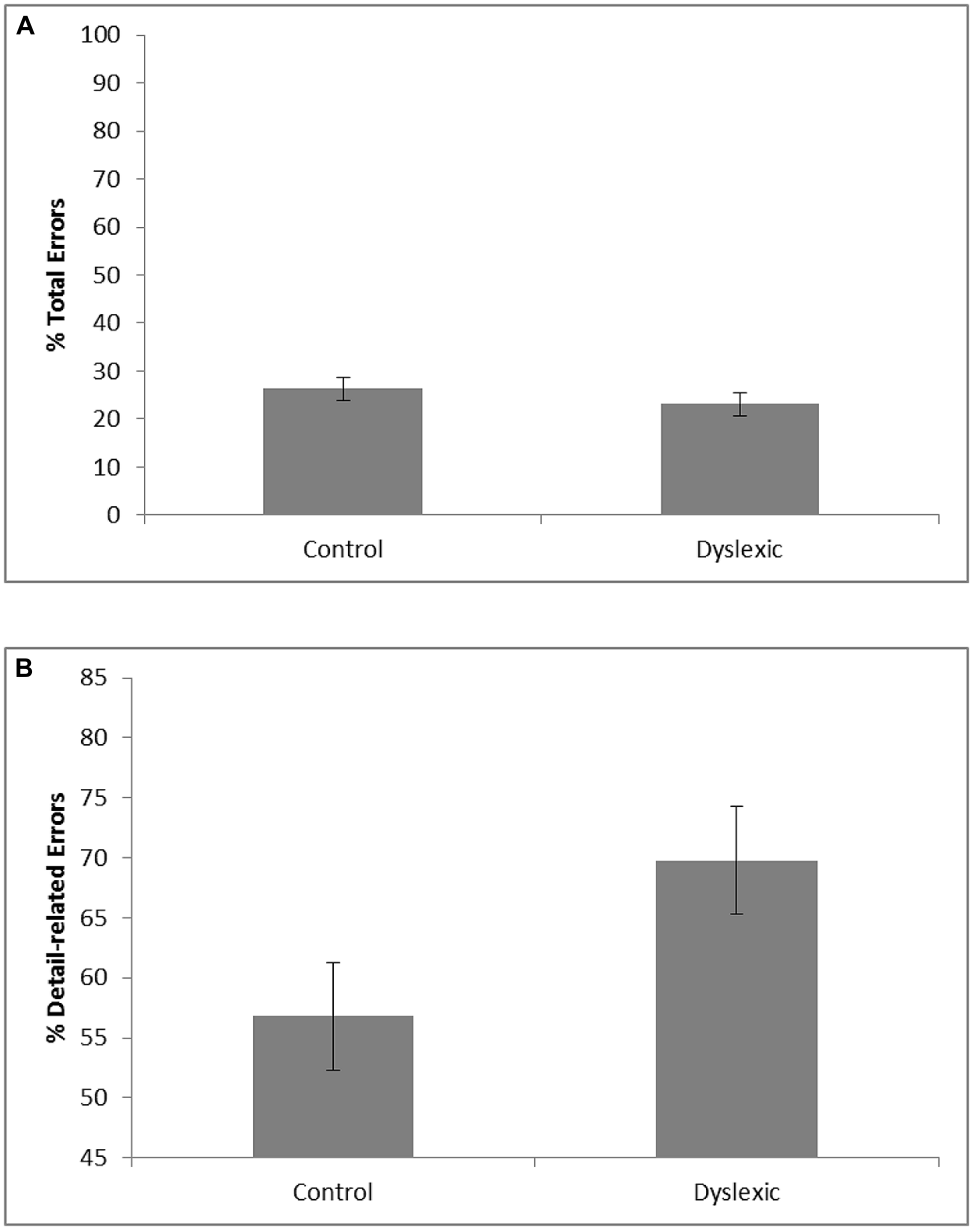
**Mean percentage of total visual memory errors (A) and mean portion of detail-related visual memory errors relative to the number of total visual memory errors (B) as a function of group (error bars represent SE)**.

The VLTM-D results showed that dyslexic children (*M* = 23.1; SE = 2.4) and controls (*M* = 26.3; SE = 2.4) did not significantly differ with respect to the total percentage of memory errors, *F* < 1. Importantly, however, dyslexics (*M* = 69.8%; SE = 4.5) and controls (*M* = 56.8%; SE = 4.5) significantly differed regarding the portion of detail-related errors, *F*(1,37) = 4.20, *p* = 0.048, $\eta_{p}^{2}$ = 0.10. Specifically, dyslexic children exhibited a greater portion of detail-related errors than controls (see **Figure 2**). The corresponding effect size for the difference (Cohen’s *d* = 0.69) reflects a medium to large effect (Cohen, 1969).

Finally, there was a marginally significant group effect on pseudo-word segmentation skills (BAKO subtest 1), *F*(1,37) = 3.87, *p* = 0.057, $\eta_{p}^{2}$ = 0.10, indicating that dyslexic children (*M* = 52; SE = 1.6) exhibited lower *T*-scores as compared to controls (*M* = 56; SE = 1.6). This finding regarding phonological skills was corroborated by a significant group effect on vowel substitution skills (BAKO subtest 2), *F*(1,37) = 22.30, *p* < 0.001, $\eta_{p}^{2}$ = 0.38 (dyslectics: *M* = 45; *SE* = 1.5, controls: *M* = 55; SE = 1.5; see **Table 1**). Even though the sample size of the dyslexic group was too small for far-reaching interpretations of correlation coefficients, it appears interesting to note that in this group the portion of detail-related errors was not correlated with any of the phonological skill measures (*r* = 0.03, *p* = 0.90 for pseudo-word segmentation and *r* = -0.02, *p* = 0.95 for vowel substitution).

Note that the obtained pattern of results regarding the crucial VLTM-D data was not dependent on the choice to include the three covariates in the statistical model (a procedure that is known to usually reduce error variance). In fact, independent samples *t*-tests revealed the same pattern of results regarding the two VLTM-D error scores [i.e., no significant difference between dyslexics and controls regarding the total percentage of memory errors, *t* < 1, but a significant group difference regarding the portion of detail-related errors, *t*(40) = 2.13; *p* = 0.039].

## DISCUSSION

Previous studies demonstrated that dyslexics exhibit degraded orthographic representations, which is evident in impaired spelling performance (Berninger et al., 2008), and deficits in verbal LTM (see Vellutino et al., 1995). Here, we hypothesized that such poor orthographic long-term representations may be associated with a general cognitive problem of representing highly detailed visual objects in LTM. Thus, we predicted a spatial resolution deficit in VLTM of dyslexic children. To test this prediction, we compared dyslexic children with age-, IQ-, and gender-matched controls regarding their performance in VLTM.

Specifically, we asked children to memorize a set of highly detailed visual objects that later had to be recognized among sets of visually similar distracters. The core result of our study is that while the overall amount of LTM errors was comparable between groups, dyslexics showed a significantly greater portion of detail-related memory errors, suggesting a specific spatial resolution deficit in VLTM of dyslexic children. Previous studies on the role of VLTM in dyslexia only yielded mixed results (ranging from normal to impaired LTM functions; see Nelson and Warrington, 1980; Bell, 1990; Watson and Willows, 1995; Menghini et al., 2010), probably because these studies did not specifically address the issue of VLTM resolution, which may be of utmost importance regarding the storage of visual word forms in the mental lexicon.

Unlike many previous studies on differences between dyslexic children and controls, we carefully controlled for gender, age, and non-verbal IQ. It is important to note that our inclusion criteria for the dyslexia group were rather liberal. Thus, the size of our present group differences in detail-related VLTM performance likely represents a conservative estimate. Given that the total number of memory errors was comparable between groups, it is interesting to note that the percentage of object-related errors (defined as the complement to the percentage of detail-related errors) is actually lower in dyslexic children. Probably, this indicates that in dyslexics more attentional weight is put on the processing of whole objects, at the expense of detail-related LTM processing.

Principally, two reasons for the association between dyslexia and impaired VLTM resolution appear conceivable. First, low VLTM resolution may be a cause of dyslexia, so that lower visual memory skills are responsible for less accurate orthographic representations. Second, low VLTM resolution may have resulted from a lack of reading exposure in dyslexics (i.e., more reading experience in non-dyslexic participants may have served as a training of overall visual resolution skills). In the latter case, the lack of visual resolution skills would most probably further impede reading acquisition, effectively amplifying both deficits. Previous research has sorted out the issue of causality by implementing longitudinal or reading-level matched designs (Goswami, 2003). For example, phonological skills in dyslexics were shown to be deteriorated even when compared to younger (reading-level matched) controls, suggesting a causal role of phonological deficits in dyslexia (Bradley and Bryant, 1983; Frith and Snowling, 1983; Olson et al., 1989; Hoeft et al., 2006), whereas magnocellular deficits (visual motion processing) in dyslexics were recently discovered to be rather a *consequence* of reduced reading exposure rather than a cause of dyslexia (Olulade et al., 2013). However, it should also be noted that even if we had employed a reading-level matched design, any lack of a significant group difference in VLTM-D would not have ruled out the possibility of a causal role of impaired VLTM-D in dyslexia (since lower memory skills in the group with younger participants would also be expected based on typical developmental trajectories of memory skills, see Gathercole, 2003). Thus, a more convincing future approach to test for causality here would be a longitudinal design in which VLTM resolution in preschoolers serves as a predictor for the development of reading problems. Based on the present data, such a study with a sufficiently large pool of participants appears a promising next step.

However, our present results are well in line with recent studies suggesting that visual processing deficits may indeed play a major causal role in developmental dyslexia. For example, Vidyasagar and Pammer (2010) suggested that a deficit in visuo-spatial attention may impair the processing of letter sequences, so that impoverished orthographic representations are fed into word recognition systems (see also Valdois et al., 2004; Bosse et al., 2007; Jones et al., 2008). Assuming that our present results regarding object memory are to some extent transferable to the language domain, our findings could suggest that the processing of degraded visual orthographic input may eventually cause somewhat impaired orthographic representations in the mental lexicon. In turn, this mechanism may instantiate a vicious circle because poor representations in LTM will make it even harder to process new orthographic input.

It is important to note that based on our study alone, we cannot finally decide whether the observed reduction in memory performance is due to encoding, maintenance, or retrieval problems. While attention-related encoding deficits are well documented in studies within the realm of the visual attention deficit account (Casco and Prunetti, 1996; Hari et al., 1999; Vidyasagar and Pammer, 1999, 2010; Facoetti et al., 2000, 2010) and the magnocellular deficit account (Eden et al., 1996), it seems puzzling why many studies in the past did not report notable deficits of visual working memory (Vellutino, 1979; Hulme, 1981; Katz et al., 1981; McDougall et al., 1994; Scanlon and Vellutino, 1997; but see Smith-Spark et al., 2003; Menghini et al., 2011), which represents the crucial gate to long-term storage. Probably, the particular tasks used in these studies may not sufficiently require the processing of visuo-spatial details.

Our results may bear interesting implications for dual-route models of word processing that assume a non-lexical route (involving grapheme-to-phoneme conversion) and a lexical route (involving orthographic lexical access via the visual word form; e.g., Coltheart et al., 2001). The first route should be especially important for beginning readers and for reading unknown words, while the lexical route is supposed to be the standard route when more experienced readers process familiar words. In line with this claim, research showed that while phonological codes are clearly activated in skilled readers’ word processing (Van Orden, 1987), the main route to lexical access appears to be the more direct visual route without any necessary involvement of phonological processing (Damian and Martin, 1998; Daneman and Reingold, 2000). Since our present results suggest that dyslexics may suffer from impoverished visual representations in LTM, this may consequently impair lexical access within the lexical route (see also Castles and Coltheart, 1993; Valdois et al., 2004; Hawelka et al., 2010). Evidence for a dedicated brain area devoted to the storage of orthographic representations in the left fusiform gyrus (i.e., the visual word form area) comes from recent neuropsychological studies (McCandliss et al., 2003; Cohen and Dehaene, 2004; Vinckier et al., 2007; Dehaene et al., 2010). Interestingly, this area is also sensitive to other complex stimuli, such as pictures (Price and Devlin, 2003), which further corroborates our claim that a word may be regarded as a special case of a complex visual object. In line with this argument, it should be noted that reading is a cultural technique too recent to involve dedicated genetic mechanisms, so that reading processes most likely rely on partial recycling of pre-existing brain structures (Dehaene and Cohen, 2007). This claim is supported by studies showing that the storage of written materials associated with the visual word form area appears to compete with the cortical representation of other complex visual objects, particularly faces (Dehaene et al., 2010).

However, it should also be noted that not all aspects of VLTM appear to be negatively affected in dyslexic patients, since the literature does not provide reports of a strong comorbidity of dyslexia and prosopagnosia, the latter representing another case of a specific failure of LTM functions regarding complex visual objects differing in small (but significant) details. Probably, the functional specialization of the corresponding brain regions in prosopagnosia (e.g., the fusiform face area) may be greater than that of the orthographic word form area (Price and Devlin, 2003).

Many previous accounts of dyslexia suggested a phonological processing deficit as a main cause. For example, problems associated with grapheme–phoneme conversion (within the non-lexical route) easily explain typical symptoms of dyslexic children, for example, error-prone and slow pronunciation during oral reading (see Hawelka et al., 2010). However, several observations suggest that phonological deficits alone may not be able to explain all facets of dyslexia (Ziegler et al., 2008). For example, it may seem puzzling why dyslexics, who typically report similar reading problems in silent as in oral reading, do not primarily process written language via the lexical route (at least during silent reading), which is supposed to be comparatively fast and should be sufficiently developed after substantial exposure to written language. One might argue that a lack of an intact phonological route during reading acquisition may have prevented the development of an intact lexical route. Nevertheless, some further characteristics of dyslexia still remain difficult to explain within the phonological deficit account alone. For example, dyslexics usually have no severe difficulties in processing oral language (e.g., Christo et al., 2009), where phonological processing should play an important role. Furthermore, the phonological deficit account cannot readily explain why some dyslexic patients appear to have no clear phonological deficits (see Vidyasagar and Pammer, 2010).

Based on these limitations of explaining dyslexia via a phonological deficit account alone, some researchers suggested to altogether abandon the idea that phonological deficits play a major causal role in dyslexia. For example, Vidyasagar and Pammer (2010) argued that phonological deficits may be the result rather than a cause of visual processing deficits. One possibility of explaining poor performance in phonological tasks may be that the activation of visual (orthographic) representations can be used to support performance in such tasks by normally developed readers (Castles et al., 2003), but not by dyslexics. However, in the light of the literature reviewed above we would rather conclude that developmental dyslexia may be too complex to be explained by just one causal factor. Instead, the term dyslexia rather seems to entail a great variety of subtypes (Heim et al., 2008, 2010; Ziegler et al., 2008), and even multiple causal factors within each dyslexic individual may come into play (e.g., Pennington, 2006). The idea of subtype variety is also supported by our observation of impaired phonological processing in our sample of dyslexic children, and, more specifically, by the lack of any correlations between detail-related visual memory skills and phonological skills in our sample of dyslexics. Thus, our present data suggest that the mainstream of research focusing on phonological processes should at least be complimented by further, more specific studies of the many complex visual processing mechanisms in dyslexics.

Taken together, our study provides novel evidence that VLTM-D (specifically, VLTM resolution) may play an important role in developmental dyslexia. Specifically, poor orthographic representations may at least partly occur due to visuo-spatial resolution deficits in LTM. Further studies are needed to explicitly address the mechanisms behind these phenomena, specifically with respect to the interplay of visual attention, long-term encoding processes, and language processing.

## Conflict of Interest Statement

The authors declare that the research was conducted in the absence of any commercial or financial relationships that could be construed as a potential conflict of interest.

## References

[B1] Bell T. K. (1990). Rapid sequential processing in dyslexic and ordinary readers. *Percept. Mot. Skills* 71 1155–1159 10.2466/pms.1990.71.3f.1155 2087370

[B2] Berninger V. W. Nielsen K. H. Abbott R. D. Wijsman E. Raskind W. (2008). Writing problems in developmental dyslexia: under-recognized and under-treated. *J. Sch. Psychol.* 46 1–21 10.1016/j.jsp.2006.11.008 18438452 PMC2344144

[B3] Bishop D. V. M. Snowling M. J. (2004). Developmental dyslexia and specific language impairment: same or different? *Psychol. Bull.* 130 858–886 10.1037/0033-2909.130.6.858 15535741

[B4] Boets B. Wouters J. Wieringen A. Ghesquière P. (2007). Auditory processing, speech perception and phonological ability in pre-school children at high-risk for dyslexia: a longitudinal study of the auditory temporal processing theory. *Neuropsychologica* 45 1608–1620 10.1016/j.neuropsychologia.2007.01.009 17303197

[B5] Bosse M. L. Tainturier M. J. Valdois S. (2007). Developmental dyslexia: the visual attention span deficit hypothesis. *Cognition* 104 198–230 10.1016/j.cognition.2006.05.009 16859667

[B6] Bradley L. Bryant P. E. (1983). Categorizing sounds and learning to read – a causal connection. *Nature* 301 419–421 10.1038/301419a0

[B7] Cain K. (2010). *Reading Development and Difficulties.* Oxford: Blackwell

[B8] Casco C. Prunetti E. (1996). Visual search of good and poor readers: effects with targets having single and combined features. *Percept. Mot. Skills* 82 1155–1167 10.2466/pms.1996.82.3c.1155 8823883

[B9] Castles A. Coltheart M. (1993). Varieties of developmental dyslexia. *Cognition* 47 149–180 10.1016/0010-0277(93)90003-E 8324999

[B10] Castles A. Davis C. Forster K. I. (2003). “Word recognition development in children: Insights from masked priming,” in *Masked Priming: State of the Art* eds Kinoshita S. Lupker S. (London: Psychology Press) 345–360

[B11] Cattell R. B. (1960). *Culture Fair Intelligence Test.* Champaign, IL: IPAT

[B12] Christo C. Davis J. Brock S. E. (2009). *Identifying, Assessing, and Treating Dyslexia at School*. New York, NY: Springer

[B13] Cohen J. (1969). *Statistical Power Analysis for the Behavioral Sciences.* New York: Academic Press

[B14] Cohen L. Dehaene S. (2004). Specialization within the ventral stream: the case for the visual word form area. *Neuroimage* 22 466–476 10.1016/j.neuroimage.2003.12.049 15110040

[B15] Coltheart M. Rastle K. Conrad P. Langdon R. Ziegler J. (2001). DRC: a dual route cascaded model of visual word recognition and reading aloud. *Psychol. Rev.* 108 204–256 10.1037/0033-295X.108.1.204 11212628

[B16] Damian M. F. Martin R. C. (1998). Is visual lexical access based on phonological codes? Evidence from a picture-word interference task. *Psychon. Bull. Rev.* 5 91–95 10.3758/BF03209461

[B17] Daneman M. Reingold E. M. (2000). “Do readers use phonological codes to activate word meanings? Evidence from eye movements,” in *Reading as a Perceptual Process,* eds Kennedy A. Radach R. Heller D. Pynte J. (Elsevier: Amsterdam) 447–473

[B18] Dehaene S. Cohen L. (2007). Cultural recycling of cortical maps. *Neuron* 56 384–398 10.1016/j.neuron.2007.10.004 17964253

[B19] Dehaene S. Pegado F. Braga L. W. Ventura P. Filho G. N. Jobert A. (2010). How learning to read changes the cortical networks for vision and language. *Science* 330 1359–1364 10.1126/science.1194140 21071632

[B20] Dehn M. J. (2010). *Long-Term Memory in Children and Adolescents.* New Jersey: John Wiley & Son. 10.1002/9781118269688

[B21] Denckla M. B. Rudel R. (1976). Rapid “automized” naming (R.A.N.): dyslexia differentiated from other learning disabilities. *Neuropsychologia* 14 471–479 10.1016/0028-3932(76)90075-0 995240

[B22] Eden G. F. Van Meter J. W. Rumsey J. M. Maisog J. M. Woods R. P. Zeffiro T. A. (1996). Abnormal processing of visual motion in dyslexia revealed by functional brain imaging. *Nature* 382 66–69 10.1038/382066a0 8657305

[B23] Facoetti A. Paganoni P. Turatto M. Marzola V. Mascetti G. G. (2000). Visuospatial attention in developmental dyslexia. *Cortex* 36 109–123 10.1016/S0010-9452(08)70840-2 10728901

[B24] Facoetti A. Trussardi A. N. Ruffino M. Lorusso M. L. Cattaneo C. Galli R. (2010). Multisensory spatial attention deficits are predictive of phonological decoding skills in developmental dyslexia. *J. Cogn. Neurosci.* 22 1011–1025 10.1162/jocn.2009.21232 19366290

[B25] Farmer M. E. Klein R. (1995). The evidence for a temporal processing deficit linked to dyslexia: a review. *Psychon. Bull. Rev.* 2 460–493 10.3758/BF03210983 24203785

[B26] Frith U. Snowling M. J. (1983). Reading for meaning and reading for sound in autistic and dyslexic children. *Br. J. Dev. Psychol.* 1 329–342 10.1111/j.2044-835X.1983.tb00906.x

[B27] Gathercole S. (2003). The development of memory. *J. Child Psychol. Psychiatry* 39 3–27 10.1017/S0021963097001753 9534084

[B28] Goswami U. (2003). Why theories about developmental dyslexia require developmental designs. *Trends Cogn. Sci.* 7 534–540 10.1016/j.tics.2003.10.003 14643369

[B29] Goswami U. Bryant P. (1990). *Phonological Skills and Learning to Read.* Hillsdale, NY: Erlbaum.

[B30] Hales G. (2008). *Dyslexia Matters.* London: Whurr.

[B31] Hari R. Valta M. Uutela K. (1999). Prolonged attentional dwell time in dyslexic adults. *Neurosci. Lett.* 271 202–204 10.1016/S0304-3940(99)00547-9 10507704

[B32] Hawelka S. Gagl B. Wimmer H. (2010). A dual-route perspective on eye movements of dyslexic readers. *Cognition* 115 367–379 10.1016/j.cognition.2009.11.004 20227686 PMC2976468

[B33] Heim S. Grande M. Pape-Neumann J. Van Ermingen M. Meffert E. Grabowska A. (2010). Interaction of phonological awareness and magnocellular processing during normal and dyslexic reading: behavioural and fMRI investigations. *Dyslexia* 16 258–282 10.1002/dys.409 20680995

[B34] Heim S. Tschierse J. Amunts K. Wilms M. Vossel S. Willmes K. (2008). Cognitive subtypes of dyslexia. *Acta Neurobiol. Exp.* 68 73–82 10.55782/ane-2008-1674 18389017

[B35] Hoeft F. Hernandez A. McMillon G. Taylor-Hill H. Martindale J. L. Meyler A. (2006). Neural basis of dyslexia: a comparison between dyslexic and nondyslexic children equated for reading ability. *J. Neurosci.* 26 10700–10708 10.1523/JNEUROSCI.4931-05.2006 17050709 PMC6674758

[B36] Huestegge L. Heim S. Zettelmeyer E. Lange-Küttner C. (2012). Gender-specific contribution of a visual cognition network to reading abilities. *Br. J. Psychol.* 103 117–128 10.1111/j.2044-8295.2011.02050.x 22229778

[B37] Hulme C. (1981). The effects of manual tracing on memory in normal and retarded-readers: some implications for multi-sensory teaching. *Psychol. Res.* 43 179–191 10.1007/BF00309828 7302088

[B38] Jones M. W. Branigan H. P. Kelly L. (2008). Visual deficits in developmental dyslexia: relationships between non-linguistic visual tasks and their contribution to components of reading. *Dyslexia* 14 95–115 10.1002/dys.345 17874457

[B39] Katz R. B. Shankweiler D. Liberman I. Y. (1981). Memory for item order and phonetic recoding in the beginning reader. *J. Exp. Child Psychol.* 32 474–484 10.1016/0022-0965(81)90109-0 7320681

[B40] Marx H. (1998). *Knuspels Leseaufgaben: Gruppenlesetest für Kinder Ende des ersten bis vierten Schuljahres*. Göttingen: Hogrefe

[B41] Mayringer H. Wimmer H. (2003). *Salzburger Lese-Screening für die Klassenstufen* 1–4 Göttingen: Hogrefe

[B42] McCandliss B. C. Cohen L. Dehaene S. (2003). The visual word form area: expertise for reading in the fusiform gyrus. *Trends Cogn. Sci.* 7 293–299 10.1016/S1364-6613(03)00134-7 12860187

[B43] McDougall S. Hulme C. Ellis A. W. Monk A. (1994). Learning to read: the role of short-term memory and phonological skills. *J. Exp. Child Psychol.* 58 112–123 10.1006/jecp.1994.1028 8064216

[B44] Menghini D. Carlesimo G. A. Marotta L. Finzi A. Vicari S. (2010). Developmental dyslexia and explicit long-term memory. *Dyslexia* 16 213–225 10.1002/dys.410 20680992

[B45] Menghini D. Finzi A. Carlesimo G. A. Vicari S. (2011). Working memory impairment in children with developmental dyslexia: is it just a phonological deficity? *Dev. Neuropsychol.* 36 199–213 10.1080/87565641.2010.549868 21347921

[B46] Nelson H. E. Warrington E. K. (1980). An investigation of memory functions in dyslexic children. *Br. J. Psychol.* 71 487–503 10.1111/j.2044-8295.1980.tb01762.x 7437673

[B47] Nicolson R. I. Fawcett A. J. (2011). Dyslexia, dysgraphia, procedural learning and the cerebellum. *Cortex* 47 117–127 10.1016/j.cortex.2009.08.016 19818437

[B48] Nicolson R. I. Fawcett A. J. Dean P. (1995). Time estimation deficits in developmental dyslexia: evidence for cerebellar involvement. *Proc. Biol. Sci.* 259 43–47 10.1098/rspb.1995.0007 7700875

[B49] Olson R. Wise B. Conners F. Rack J. Fulker D. (1989). Specific deficits in component reading and language skills: genetic and environmental influences. *J. Learn. Disabil.* 22 339–348 10.1177/002221948902200604 2738467

[B50] Olulade O. A. Napoliello E. M. Eden G. F. (2013). Abnormal visual motion processing is not a cause of dyslexia. *Neuron* 79 180–190 10.1016/j.neuron.2013.05.002 23746630 PMC3713164

[B51] Pennington B. F. (2006). From single to multiple deficit models of developmental disorders. *Cognition* 101 385–413 10.1016/j.cognition.2006.04.008 16844106

[B52] Price C. J. Devlin J. T. (2003). The myth of the visual word form area. *Neuroimage* 19 473–481 10.1016/S1053-8119(03)00084-3 12880781

[B53] Ramus F. (2003). Developmental dyslexia: specific phonological deficit or general sensorimotor dysfunction? *Curr. Opin. Neurobiol.* 13 212–218 10.1016/S0959-4388(03)00035-7 12744976

[B54] Scanlon D. M. Vellutino F. R. (1997). A comparison of the instructional backgrounds and cognitive profiles of poor, average, and good readers who were initially identified as at risk for reading failure. *Sci. Stud. Read.* 1 191–215 10.1207/s1532799xssr0103_2

[B55] Smith-Spark J. H. Fisk J. E. Fawcett A. J. Nicolson R. I. (2003). Investigating the central executive in adult dyslexics: evidence from phonological and visuospatial working memory performance. *Eur. J. Cogn. Psychol.* 15 567–587 10.1080/09541440340000024

[B56] Snowling M. J. (2000). *Dyslexia.* Oxford: Blackwell

[B57] Snowling M. J. (2001). From language to reading and dyslexia. *Dyslexia.* 7 37–46 10.1002/dys.185 11305230

[B58] Stein J. (2001). The magnozellular theory of developmental dyslexia. *Dyslexia* 7 12–36 10.1002/dys.186 11305228

[B59] Stein J. Walsh V. (1997). To see but not to read; the magnocellular theory of dyslexia. *Trends Neurosci.* 20 147–152 10.1016/S0166-2236(96)01005-3 9106353

[B60] Stock C. Marx P. Schneider W. (2003). *BAKO 1–4: Basiskompetenzen für Lese-Rechtschreibleistungen*. Beltz: Göttingen

[B61] Studdert-Kennedy M. (2002). Deficits in phoneme awareness do not arise from failures in rapid auditory processing. *Read. Writ.* 15 5–14 10.1023/A:1013812219382

[B62] Tallal P. Miller S. Fitch R. H. (1993). Neurobiological basis of speech: a case for the preeminence of temporal processing. *Ann. N. Y. Acad. Sci.* 682 27–47 10.1111/j.1749-6632.1993.tb22957.x 7686725

[B63] Thambirajah M. S. (2011). *Developmental Assessment of the School-aged Child with Developmental Disabilities.* London: Jessica Kingsley Publishers

[B64] Valdois S. Bosse M. L. Tainturier M. J. (2004). The cognitive deficits responsible for developmental dyslexia: review of evidence for a selective visual attentional disorder. *Dyslexia* 10 339–363 10.1002/dys.284 15573964

[B65] Van Orden G. C. (1987). A ROWS is a ROSE: spelling, sound and reading. *Mem. Cogn.* 15 181–198 10.3758/BF03197716 3600258

[B66] Vellutino F. R. (1979). *Dyslexia: Theory and Research.* Cambridge, MA: MIT Press

[B67] Vellutino F. R. Fletcher J. M. Snowling M. J. Donna M. S. (2004). Specific reading disability (dyslexia): what have we learned in the past four decades? *J. Child Psychol. Psychiatry* 45 2–40 10.1046/j.0021-9630.2003.00305.x 14959801

[B68] Vellutino F. R. Scanlon D. M. Spearing D. (1995). Semantic and phonological coding in poor and normal readers. *J. Exp. Child Psychol.* 59 76–123 10.1006/jecp.1995.1004 7876770

[B69] Vidyasagar T. R. Pammer K. (1999). Impaired visual search in dyslexia relates to the role of the magnocellular pathway in attention. *Neuroreport* 10 1283–1287 10.1097/00001756-199904260-00024 10363940

[B70] Vidyasagar T. R. Pammer K. (2010). Dyslexia: a deficit in visuo-spatial attention, not in phonological processing. *Trends Cogn. Sci.* 14 52–63 10.1016/j.tics.2009.12.003 20080053

[B71] Vinckier F. Dehaene S. Jobert A. Dubus J. P. Sigman M. Cohen L. (2007). Hierarchical coding of letter strings in the ventral stream: dissecting the inner organization of the visual word-form system. *Neuron* 55 143–156 10.1016/j.neuron.2007.05.031 17610823

[B72] Watson C. Willows D. M. (1995). Information-processing patterns in specific reading disability. *J. Learn. Disabil.* 28 216–231 10.1177/002221949502800404 7738434

[B73] Weiss R. H. (1998). *Grundintelligenztest Skala 2 (CFT 20).* Göttingen: Hogrefe

[B74] Wolf M. Bowers P. G. (1999). The double deficit hypothesis for the developmental dyslexia. *J. Educ. Psychol.* 91 1–24 10.1037/0022-0663.91.3.415

[B75] Ziegler J. C. Castel C. Pech-Georgel C. George F. Alario F. Perry C. (2008). Developmental dyslexia and the dual route model of reading: simulating individual differences and subtypes. *Cognition* 107 151–178 10.1016/j.cognition.2007.09.004 17959161

